# Value of Spinal Cord Diffusion Imaging and Tractography in Providing Predictive Factors for Tumor Resection in Patients with Intramedullary Tumors: A Pilot Study

**DOI:** 10.3390/cancers16162834

**Published:** 2024-08-13

**Authors:** Corentin Dauleac, Timothée Jacquesson, Carole Frindel, Nathalie André-Obadia, François Ducray, Patrick Mertens, François Cotton

**Affiliations:** 1 Hospices Civils de Lyon, Hôpital Neurologique et Neurochirurgical Pierre Wertheimer, Service de Neurochirurgie de la Moelle Spinale et des Nerfs Périphériques, 69002 Lyon, France; patrick.mertens@chu-lyon.fr; 2 Faculté de Médecine Lyon-Est, Université Claude Bernard Lyon I, 69100 Villeurbanne, France; timothee.jacquesson@chu-lyon.fr (T.J.); francois.ducray@chu-lyon.fr (F.D.); francois.cotton@chu-lyon.fr (F.C.); 3 Laboratoire CREATIS, CNRS UMR 5220, Inserm U1296, INSA Lyon, 69100 Villeurbanne, France; carole.frindel@creatis.insa-lyon.fr; 4 Hospices Civils de Lyon, Hôpital Neurologique et Neurochirurgical Pierre Wertheimer, Service de Neurochirurgie Crânienne, 69002 Lyon, France; 5 Hospices Civils de Lyon, Hôpital Neurologique et Neurochirurgical Pierre Wertheimer, Service de Neurologie Fonctionnelle et Electrophysiologie, 69002 Lyon, France; nathalie.obadia-andre@chu-lyon.fr; 6 Hospices Civils de Lyon, Hôpital Neurologique et Neurochirurgical Pierre Wertheimer, Service de Neuro-Oncologie, 69002 Lyon, France; 7 Hospices Civils de Lyon, Centre Hospitalier Lyon Sud, Service de Radiologie, 69002 Lyon, France

**Keywords:** spinal cord, tractography, high angular resolution diffusion imaging, spinal cord tumors, predictive factor

## Abstract

**Simple Summary:**

This pilot study aimed to highlight the value of spinal cord diffusion imaging and tractography in providing predictive factors for tumor resection in patients with intramedullary tumors. Tensor metrics provided predictive factors of spinal cord damage with a high correlation between fractional anisotropy, mean diffusivity, and radial diffusivity, and suggested changes in microstructural architecture, axonal density, and myelinated fibers of the spinal cord. Spinal cord tractography also provided predictive factors for tumor resection by helping to perform histological tumor differentiation and differentiating spinal tracts and, thus, defining relationships between spinal cord tissue and tumor; all these elements argue in favor of tumor resectability.

**Abstract:**

This pilot study aimed to investigate the interest of high angular resolution diffusion imaging (HARDI) and tractography of the spinal cord (SC) in the management of patients with intramedullary tumors by providing predictive elements for tumor resection. Eight patients were included in a prospective study. HARDI images of the SC were acquired using a 3T MRI scanner with a reduced field of view. Opposed phase-encoding directions allowed distortion corrections. SC fiber tracking was performed using a deterministic approach, with extraction of tensor metrics. Then, regions of interest were drawn to track the spinal pathways of interest. HARDI and tractography added value by providing characteristics about the microstructural organization of the spinal white fibers. In patients with SC tumors, tensor metrics demonstrated significant changes in microstructural architecture, axonal density, and myelinated fibers (all, *p* < 0.0001) of the spinal white matter. Tractography aided in the differentiation of tumor histological types (SC-invaded vs. pushed back by the tumor), and differentiation of the spinal tracts enabled the determination of precise anatomical relationships between the tumor and the SC, defining the tumor resectability. This study underlines the value of using HARDI and tractography in patients with intramedullary tumors, to show alterations in SC microarchitecture and to differentiate spinal tracts to establish predictive factors for tumor resectability.

## 1. Introduction

Spinal cord tumors are rare, accounting for 2% to 14% of all central nervous system neoplasms in adults [1]. However, spinal cord tumors present major neurosurgical challenges due to the long and thin structure of the spinal cord, as well as the high density of white matter fibers in a very small volume. For these reasons, intramedullary tumors can be highly symptomatic, causing motor weakness, sensitivity, and/or bladder and sexual disorders. According to clinical symptoms, surgical management remains largely the gold standard for these tumors [2,3,4,5]. As intramedullary surgical procedures can induce significant neurological morbidities [6,7,8], accurate preoperative imaging is essential to differentiate between tumor tissue and spinal tracts [9], thereby potentially minimizing the risk of postoperative neurological deficits.

Improvements in spinal cord diffusion tensor imaging over the past decade, with the use of high angular resolution diffusion imaging (HARDI), have enhanced the accuracy and reliability of fiber tracking, and highlighted the potential key role of spinal cord tractography compared to conventional MR sequences [10,11,12]. As demonstrated for the central and peripheral central nervous system, HARDI provides valuable insights into the microstructural architecture of neural tissue [13]. Spinal cord tractography enables the visualization of the orientation and integrity of white matter fibers, which can be crucial in identifying the displacement or infiltration of spinal tracts by intramedullary tumors [14], thus providing additional information for preoperative planning [15]. However, to date, authors performing spinal cord tractography have not provided any information on the anatomical relationships of spinal cord tumors with white fiber tracts. To the best of our knowledge, spinal tract differentiation has never been established, though it could have a major impact on the surgical management of patients with intramedullary tumors.

Based on the potential of HARDI to illuminate the intricate pathways and connectivity [16] within the spinal cord [17], this pilot study first aimed to investigate the microstructural integrity of the spinal cord in patients with intramedullary tumors by analyzing tensor metrics and 3D tractography rendering. Secondly, this study aimed to assess the potential key role of spinal cord tractography in surgical planning by accurately providing the relationship between tumor and spinal tracts, thus providing predictive factors for tumor resectability.

## 2. Materials and Methods

The study was conducted in accordance with the 1964 Helsinki Declaration, and approved by the Ethics Committee (Comité de Protection des Personnes Sud-Méditerranée II, 2021-A02277-34) and by the the national data protection committee (Commission nationale de l’informatique et des libertés, MR0001, 10/28/2021for studies involving humans. Written informed consent was obtained from all participants.

### 2.1. Demographics

Eight patients participated in this prospective, single-center, pilot study conducted in our university hospital. Patients with cervical or cervicothoracic spinal cord tumors were included. Exclusion criteria included MRI contraindication.

Moreover, 49 healthy subjects without previous neurological or neurosurgical history at the brain, spine, and spinal cord levels, and without dental implants or braces (to minimize MRI artifacts) were included and used as a reference to compare tensor metrics at each spinal level between patients and healthy subjects.

### 2.2. Neurological Evaluation

Clinical assessment included examination of sensory, motor, bladder, bowel, and sexual functions, as well as gait. The modified McCormick scale was used to assess neurological functions [7].

Electrophysiological assessment included motor evoked potentials (MEP) and somatosensory evoked potentials (SSEP), and was reviewed by 2 electrophysiologists. The neurophysiological scale proposed by Dauleac et al. [18] was used to assess the neurophysiological functions of the upper and lower limbs; where grade A signifies that MEP and SSEP were normal, grade B denotes the presence of isolated motor and/or somesthetic segmental alteration, grade C signifies that there was a lengthening of the motor or somesthetic central conduction time, grade D indicates a delay in both motor and somesthetic central conduction times, grade E indicates a prolonged central conduction time for either the motor or somesthetic modality accompanied by the abolition of the other modality response, and grade F indicates the abolition of both motor and somesthetic evoked responses.

### 2.3. Data Acquisition

Patients were positioned with the neck in a neutral position to have the cervical spinal cord as straight as possible, in line with the brainstem. If cervical lordosis was seen on the initial survey, a cushion was placed under the subject’s head, or the subject was asked to tilt his/her head towards his/her chest, to standardize cervical lordosis angles.

#### 2.3.1. High Angular Resolution Diffusion Imaging (HARDI)

Diffusion images were obtained using a 3T Ingenia Elition MRI machine (Philips Medical Systems, Eindhoven, Netherlands) equipped with 32-channel coils and utilizing zonally magnified oblique multi-slice (ZOOM) echo planar imaging. The ZOOM method leverages the concept of inner volume imaging, allowing for the selective excitation and refocusing of a narrow field of view (224 × 56 × 40 mm), whilst minimizing signals originating from outside the targeted field of view [19]. This approach reduces geometric distortions, including those caused by field inhomogeneity, susceptibility, and motion artifacts, without aliasing within the field of view. The imaging parameters used were as follows: b-value: 0 and 1000 s/mm^2^, TE/TR: 64/3220 ms, isotropic voxel size 2 × 2 × 2 mm, no slice gap, coronal plane, with fat suppression (SPIR). The acquisition volume was oriented along the axis of the spinal cord. The sequence was first acquired in 32 directions with the right–left phase-encoding direction in 3 min 45 s, and then in 32 directions with the left–right phase-encoding direction, without in-plane acceleration.

#### 2.3.2. Anatomical Imaging

Sagittal T2-weighted imaging was acquired using Turbo Spin Echo, with the following parameters: field of view 300 × 31 × 160 mm, voxel size 0.8 × 0.95 × 2 mm, no slice gap, TE/TR: 120/3500 ms, in 2 min 27 s.

### 2.4. Data Processing

#### 2.4.1. Distortion Corrections

The acquisition of HARDI images with opposed phase encoding directions allowed the reduction in inhomogeneity distortions [20] using the functional magnetic resonance imaging of the brain Software Library (FSL) [21] topup [22], implemented in DSI Studio, version Aug 2022 (used afterward) [12]. Artifacts generated by eddy currents produced during diffusion-encoding gradient application were corrected using FSL eddy, implemented in DSI Studio. Then, each diffusion-weighted image was linearly registered to the b0-image using mutual information to correct motion artifacts.

#### 2.4.2. Fiber Tracking

The working mask was dilated and adjusted to include the whole spinal cord, but to exclude the non-nervous system, which potentially represented noise in the images.

Tractography was performed using DSI Studio software (version Aug 2022) [12]. DSI Studio provides a deterministic fiber-tracking algorithm based on a generalized q-sampling imaging approach (HARDI) [23], improving accuracy [12]. Up-sampling by 2 was conducted to achieve a high spatial resolution. The tractography algorithm used the following parameters: step size = 0.1 mm, fiber length = 10 to 1000 mm, angular threshold = 90°. A total of 10,000,000 seeds were calculated. Fibers were displayed as tubes with a 0.10 mm diameter. At this step, no region of interest (ROI) was drawn, and a “full” spinal cord tractography rendering was obtained [24]. Then, the spinal cord fiber tracking previously obtained was classified using the Setzer et al. classification [25], where type 1 indicates that fibers did not enter the lesion, type 2 indicates that fibers crossed the lesion but most of the lesion was free of fibers, and type 3 indicates that most of the tumor volume contained fibers or the tumor led to the destruction of fibers.

#### 2.4.3. ROI Design

Then, ROIs were drawn using T2-weighted imaging, secondly merged on an FA color map, on (1) the pyramids (of the medulla oblongata) to track corticospinal tracts (CSTs), (2) the (superior and inferior) cerebellar peduncles to track the anterior and the posterior spinocerebellar tracts, and (3) the medial lemniscus to track somatosensory pathways. Then, each tract was classified using the Czernicki et al. classification [26], where stage 0 indicates a normal tract, stage I indicates that the tract was intact but displaced from its normal anatomical location, stage II indicates that the tract was partially disintegrated by tumors, and stage III indicates that the tract was completely disintegrated by tumors.

#### 2.4.4. Spinal Cord Segmentation and Data Extraction

Registration of spinal cord tractography and sagittal T2-weighted imaging was then automatically achieved. This allowed us to appreciate the relationship between tracts of interest and anatomical images, and to segment the spinal cord. A new region was drawn at each spinal level, taking care not to draw the region at voxels overlapping the cord and cerebrospinal fluid to reduce partial volume effects. After spinal level segmentation, tensor metrics (fractional anisotropy (FA), mean diffusivity (MD), axial diffusivity (AD), and radial diffusivity (RD)) were extracted at each spinal cord level. FA measures the degree of anisotropy and represents a summary measure of microstructural axonal integrity [27]; MD refers to the mean apparent diffusion coefficient values [27] and is sensitive to edema and cell infiltration; AD measures the rate of water molecules diffusing along the primary diffusion direction and is sensitive to axonal caliber [28], axonal density, and axonal injury [29]; and RD measures the rate of water molecules diffusing perpendicular to the primary diffusion direction, and is sensitive to myelination [28].

### 2.5. Statistical Analysis

Descriptive statistics were used to summarize the demographic and clinical characteristics of the study population. Continuous variables were reported as mean ± standard deviation, while categorical variables were reported as frequencies and percentages. Associations between tractography classification (i.e., Setzer classification), clinical status, and neurophysiological status were analyzed using either the Chi-square test or Fisher’s exact test, as appropriate. Tensor metric comparisons between patients with intramedullary tumors and healthy subjects were evaluated using independent t-tests. Within the patient group, the correlation between FA, MD, and RD values within the tumor was assessed using Spearman’s rank correlation coefficient (ρ). A *p*-value < 0.05 was considered statistically significant. Statistical analyses were conducted using a web-based statistical tool (https://biostatgv.sentiweb.fr (accessed on 18 June 2024)).

## 3. Results

Eight patients with intramedullary tumors were included in this pilot study. There were four (50.0%) females, and the mean age of the population was 50 ± 11.9 years. According to the modified McCormick scale, two (25.0%) patients were grade I, four (50.0%) were grade II, and two (25.0%) were grade III. According to the neurophysiological scale, two (25.0%) patients were grade A, one (12.5%) was grade B, three (37.5%) were grade C, one (12.5%) was grade D, and one (12.5%) was grade E (Table 1).

Six (75.0%) tumors were located in the cervical cord, two (25.0%) in the high portion of the thoracic cord, and one of them had a syrinx in the whole cervical cord. Tumors were homogenously enhanced in two (25.0%) patients, while only tumor walls were enhanced in two (25.0%) patients, and not enhanced in four (50.0%) patients (Table 2). Tumor diagnosis were represented by ependymoma (n = 5, 62.5%), cavernous hemangioma (n = 2, 25.0%), and astrocytoma (n = 1, 12.5%).

### 3.1. Tensor Metrics

Compared to healthy subjects, and considering spinal levels, the FA was significantly decreased (0.24 ± 0.12 vs. 0.55 ± 0.05, *p* < 0.0001), MD was significantly increased (1.92 ± 0.36 vs. 1.25 ± 0.14 10^−3^ mm^2^/s, *p* = 0.0001), RD was significantly increased (1.70 ± 0.44 vs. 0.83 ± 0.15 mm^2^/s, *p* < 0.0001), and AD was not decreased (2.36 ± 0.26 vs. 2.10 ± 0.15), at the tumor levels, in patients with intramedullary tumors. There was a negative correlation between FA and MD values in the tumors (ρ = −0.81, *p* = 0.002), and between FA and RD values in the tumors (ρ = −0.89, *p* = 0.0002).

In patients with cavernous hemangioma, changes in FA, MD, and RD were observed only at the tumor level (while other levels were free of tensor metrics abnormalities, compared to the healthy population), while in patients with ependymoma and astrocytoma, changes in FA, MD, and RD were also observed above and below the tumor level (Supplementary Materials).

### 3.2. Tractography Rendering

In all cases, spinal cord tractography rendering was considered helpful in the decision-making process for management. Based on the spinal cord tractography rendering, four (50.0%) were type 1, three (37.5%) were type 2, and one (12.5%) was type 3 according to the Setzer classification (Table 2). The infiltration of spinal tracts by the tumor (i.e., the Setzer classification) was significantly associated with the clinical status (modified McCormick scale, *p* = 0.03), and the neurophysiological status (*p* = 0.04).

**Figure 1:**
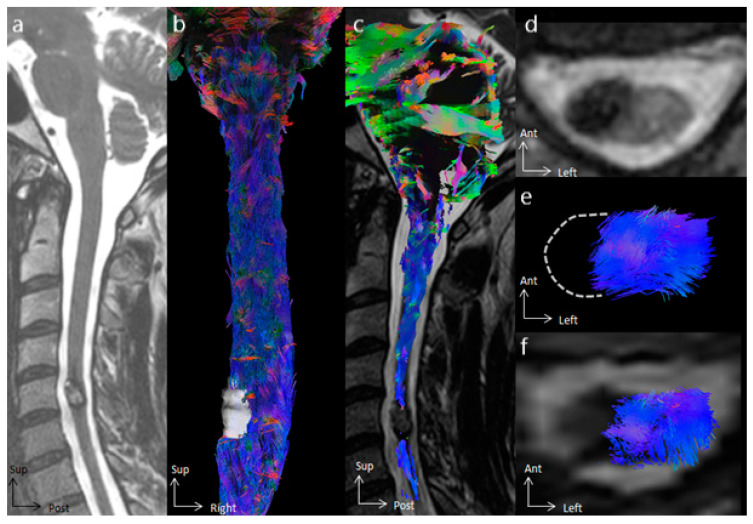
Spinal cord tractography in patient 6, with a cavernous hemangioma. (**a**) Sagittal T2-weighted image showing a cavernous hemangioma at the C5 spinal level, surrounded by hemosiderin crown in a hyposignal T2-weighted sequence. (**b**) Ventral view of the spinal cord tractography with 3D rendering of the cavernous hemangioma (in white). (**c**) Overlay of the spinal cord tractography on the sagittal T2-weighted MR images, demonstrating that no fibers were within the tumor. (**d**) Axial T2-weighted images showing a left exophytic intramedullary cavernous hemangioma. (**e**) Axial view of spinal cord tractography at the C5 level. (**f**) Overlay of the spinal cord tractography on the axial T2-weighted MR images, showing that spinal fibers were pushed back and compressed by the cavernous hemangioma, without fibers within the lesion.

**Figure 2:**
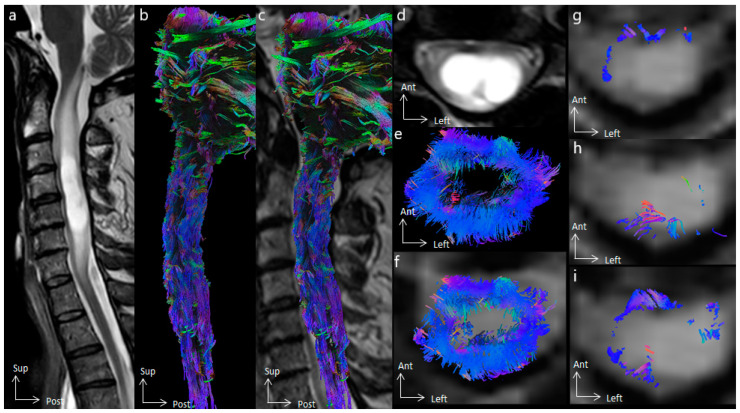
Spinal cord tractography in patient 5, with an ependymoma. (**a**) Sagittal T2-weighted image showing cystic C3–C4 intramedullary tumor, whose cystic walls are enhanced after gadolinium injection. (**b**) Lateral view of the spinal cord tractography. (**c**) Overlay of the spinal cord tractography on the sagittal T2-weighted MR images, suggesting lateral loss of fibers at the C3 spinal level. (**d**) Axial T2-weighted images showing a doubled-lobed cystic tumor. (**e**) Axial view of spinal cord tractography at the C4 level. (**f**) Overlay of the spinal cord tractography on the axial T2-weighted MR images, showing that spinal fibers were compressed by the tumor on the surface of the spinal cord, and suggesting ependymoma. (**g**) Overlay of the corticospinal tract on the axial T2-weighted MR images, compressed ventrally. (**h**) Overlay of the dorsal columns on the axial T2-weighted MR images, compressed dorsally, and within the tumor on the left side. (**i**) Overlay of the spinocerebellar tracts on the axial T2-weighted MR images.

**Figure 3:**
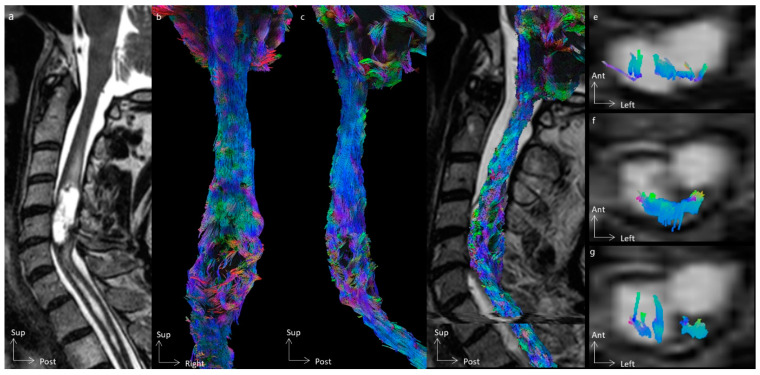
Spinal cord tractography in patient 3, with an ependymoma, grade II. (**a**) Sagittal T2-weighted image showing a cystic C4–C5 intramedullary tumor, whose cystic walls are enhanced after gadolinium injection. (**b**,**c**) Ventral and lateral views of the spinal cord tractography, showing white fiber disorganization at the tumor level. (**d**) Overlay of the spinal cord tractography on the sagittal T2-weighted MR images. (**e**) Overlay of the corticospinal tract on the axial T2-weighted MR images, within the tumor. (**f**) Overlay of the dorsal columns on the axial T2-weighted MR images, compressed dorsally, and within the tumor on the left side. (**g**) Overlay of the spinocerebellar tracts on the axial T2-weighted MR images.

**Figure 4:**
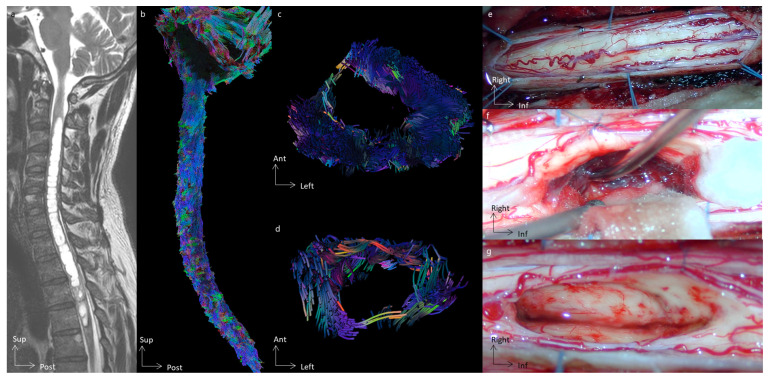
Spinal cord tractography in patient 2, with an ependymoma. (**a**) Sagittal T2-weighted image showing an intramedullary tumor at the T3 spine level, surrounded by a voluminous syrinx extending up to C3. (**b**) Sagittal view of the spinal cord tractography with 3D rendering. (**c**) Axial view of spinal cord tractography at the level of the syrinx (C5 level). (**d**) Axial view of spinal cord tractography at the tumor level (T3 level). (**e**) Operative view of the spinal cord before the opening of the median dorsal sulcus. (**f**) Operative view once the spinal cord is open and dorsal tracts gently retracted, allowing for a safe removal of the tumor. A cleavage plane was found, and as demonstrated by the 3D tractography rendering, the tumor did not invade the spinal cord. (**g**) Operative view demonstrating a gross total resection of the tumor.

**Figure 5:**
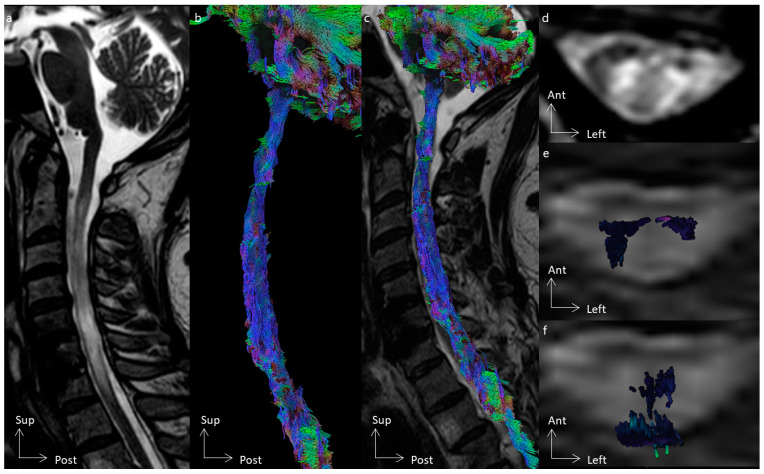
Spinal cord tractography in patient 1, with an astrocytoma. (**a**) Sagittal T2-weighted image showing a T2-weighted hypersignal at the C5–C6–C7 spine levels. (**b**) Sagittal view of the spinal cord tractography with 3D rendering. (**c**) Overlay of the spinal cord tractography on the sagittal T2-weighted MR images. (**d**) Axial T2-weighted images showing a diffuse T2-weighted hypersignal within the intramedullary tumor. (**e**) Overlay of the corticospinal tract on the axial T2-weighted MR images, within the tumor. (**f**) Overlay of the dorsal columns on the axial T2-weighted MR images, within the tumor and also compressed dorsally.

In all cases, tractography added evidence for the differentiation of the histological types of spinal cord tumors. All cases of cavernous hemangioma were classified as score 1 according to the Setzer classification, and spinal white fibers were being pushed back medially (Figure 1), as with extrinsic compression. Ependymomas were classified as scores 1 and 2 according to Setzer classification, and spinal white fibers were pushed back superficially within the spinal cord (Figure 2, Figure 3 and Figure 4), while the case of astrocytoma (Figure 5) was classified as score 3 according to the Setzer classification, and tumor invaded spinal white fibers (without pushing them back). The infiltration (or lack thereof) of spinal tracts by the tumor (i.e., the Setzer classification) was significantly associated with the histological type (*p* = 0.03).

Differentiation of spinal tracts was achieved in all subjects, except one (subject 2) who had a cervical syrinx. After differentiation of motor, sensitive, and spinocerebellar pathways, 11 (52.4%) tracts were stage I, 5 (23.8%) tracts were stage II, and 5 (23.8%) tracts were stage III according to the Czernicki classification (Table 2). The infiltration of corticospinal tracts (i.e., Czernicki classification) was not associated with MEP abnormalities (*p* = 0.4), while the infiltration of dorsal columns was significantly associated with SSEP abnormalities (*p* = 0.02).

## 4. Discussion

In conjunction with clinical and electrophysiological assessments, this pilot study underlines the value of using HARDI and spinal cord tractography in patients with intramedullary tumors, to demonstrate alterations in spinal cord microarchitecture and differentiate spinal tracts to establish predictive factors for tumor resectability.

### 4.1. Spinal Cord Microarchitecture

HARDI provided advanced pieces of information about the microstructural characteristics of the spinal cord. The decrease in FA observed in all patients with intramedullary tumors (at the tumor level, and sometimes above and below) gave information concerning microarchitectural aspects of the spinal tracts and demonstrated major alterations, which cannot always be visualized on anatomical MR images. These changes in the spinal cord tissue are the result of a reduced restriction of water motion within the spinal cord lesion. Moreover, an increase in MD was associated with vasogenic edema, and correlated with observations made on T2-weighted images. In line with our results finding a correlation between FA and MD values, Zhao et al. suggested that these metrics might be markers for the evaluation of the severity of damage [30]. In addition, the present study showed a correlation between FA and RD values, suggesting that the microarchitectural changes produced by the tumor induced a demyelination process. Although this has yet to be confirmed, tensor metrics may define prognostic markers in patients with spinal cord tumors.

### 4.2. The Added Value of Tractography

Tractography rendering provided visual information about the microstructural alteration of the spinal cord white matter. The classification introduced by Setzer et al. [25] allowed the categorization of tumors from “safely resected” (i.e., not an infiltrative tumor), to “unsafely resected” (i.e., infiltrative tumor). Information given by the tractography rendering added useful help in the decision-making process for surgical management. In addition to the anatomical images, tractography also provided arguments to suggest the anatomopathological subtypes of tumors [30,31], which can add to the neurosurgeon’s prediction value of tumor resectability [25]. Considering the prognostic factors of intramedullary surgery (which are the preoperative neurological status, and the histological type of the tumors) [8,32], spinal cord tractography appeared to be an important and useful tool for potentially helping to define predictive factors for tumor resection.

Moreover, the differentiation of spinal tracts provided preoperative planning to the specialized spinal cord neurosurgeon. Preoperative surgical planning represented an important added value by allowing the neurosurgeon to know the location of each tract of interest (i.e., motor, sensitive, and spinocerebellar pathways) and its relationship with the spinal cord tumor, potentially preventing and decreasing postoperative neurological morbidity [15]. This preoperative planning potentially could influence the surgical strategy in a biopsy, sub-total resection, and gross total resection, according to perioperative findings (presence or not of a cleavage plane), and results of the intraoperative neurophysiological monitoring. In this way, tractography did not seek to replace the clinical expertise of the neurosurgeon but rather to offer an additional tool to potentially optimize surgical outcomes.

### 4.3. Clinical and Neurophysiological Association

In this study, a significant association between clinical and electrophysiological outcomes and spinal cord infiltration detected through spinal cord tractography was observed. Patients presenting with severe deficits exhibited pronounced disruptions in the motor and sensitive pathways, as visualized on 3D tractography rendering. These disruptions were paralleled by alterations in electrophysiological parameters. Czernicki et al. also found a close relationship between corticospinal tract alterations in tractography and MEP recordings [26], reflecting the high precision and interest of diffusion tensor imaging in spinal cord lesions. The concordance between the tractography findings and the electrophysiological abnormalities suggests that diffusion tensor imaging and the associated tractography could serve as a reliable tool to explore spinal cord damage.

### 4.4. Strengths, Limitations, and Future Research

This pilot study presented a step-by-step pipeline from data acquisition to tractography rendering for patients with spinal cord tumors, for those intending to use this tool in clinical routines [24]. Although the small size and complex architecture of the spinal cord represent significant challenges for obtaining high-resolution images, the protocol proposed herein significantly decreased distortions to obtain a high-quality image [33], increasing the signal-to-noise ratio and accuracy of diffusion metrics. Moreover, the short scan time used herein allowed for decreased motion artifacts and is highly adapted to clinical practice. In addition, spinal cord tractography holds considerable promise for advancing the diagnosis [14,25], surgical planning [15,34], and prognostic evaluation of intramedullary tumors, and probably could be an important tool to increase postoperative neurological outcomes, although this remains to be demonstrated.

Nevertheless, this study had some limitations. The sample size of this study is small. Patients with mid- or lower thoracic cord tumors were not included due to the increase in motion artifacts with the acquisition of diffusion images of the thoracic cord (physiological motions such as breathing and cardiac pulsations), which potentially impacts the clinical usefulness of this methodology. Moreover, due to the heterogeneous tissue composition (tumor, edema, syrinx), the differentiation between tumoral tissue, peritumoral edema, and normal spinal cord tissue can be challenging, leading to potential inaccuracies in assessing tumor margins and the involvement of surrounding tracts. Tumors can disrupt the normal architecture of spinal tracts, complicating the delineation of fiber pathways. These limitations underscore the need for rigorous validation studies to enhance the reliability and utility of HARDI and tractography in patients with spinal cord tumors.

Future directions include improvements in magnetic resonance imaging hardware, such as higher field strengths, which are expected to significantly increase the resolution and signal-to-noise ratio of spinal cord imaging [35]. As previously demonstrated in peripheral nerves (which presented different diffusion properties according to nerve compartments [36]), ultra-high-field MRI could appreciate precise mapping of the complex fiber architecture within and around spinal cord tumors, enabling better differentiation between tumors, edematous regions, and intact spinal pathways [37]. Integration of spinal cord tractography with other imaging modalities, such as functional MRI [38], could offer a more comprehensive assessment of the architecture and function of spinal tracts and could represent a major innovation. Continued research and interdisciplinary collaboration will be crucial in translating these innovations into clinical practice, ultimately improving patient outcomes.

## 5. Conclusions

This pilot study advocates for the routine inclusion of spinal cord tractography in the diagnostic and monitoring protocols for patients with spinal cord tumors. Furthermore, 3D tractography rendering associated with clinical and electrophysiological assessments could enhance the precision of preoperative planning and prognosis, thus aiming to contribute to improved patient outcomes.

## Figures and Tables

**Table 1:** Clinical and electrophysiological assessments.

Subjects	Sex	Age	Clinical Assessment	Electrophysiological Assessment			Tumor Diagnosis
			Modified McCormick Scale	MEP	SSEP	Grade	
1	F	54	III	iCCT (UL, LLL) + RAR (UL)	iCCT (RUL) + AbR (LUL, LL)	E	Astrocytoma
2	M	44	II	normal	normal	A	Ependymoma
3	F	66	III	RAR (RUL)	AbR (LL)	D	Ependymoma
4	M	59	I	normal	AbR (LL)	B	Ependymoma
5	M	57	II	normal	iCCT (LUL)	C	Ependymoma
6	M	28	II	iCCT (RUL, RLL) + RAR (RUL, RLL)	normal	C	Cavernous hemangioma
7	F	51	II	iCCT (LUL) + RAR (LUL)	normal	C	Cavernous hemangioma
8	F	41	I	normal	normal	A	Ependymoma

AbR: abolition of the response, iCCT: increased central conduction time, LL: lower limbs, LLL: left lower limb, LUL: left upper limb, MEP: motor evoked potential, RAR: reduction of the amplitude of the response, RLL: right lower limb, RUL: right upper limb, SSEP: somatosensory evoked potential, UL: upper limbs.

**Table 2:** Radiological assessments.

Subjects	MR Features				Tractography			
	Location	Gadolinium Enhancement	Syrinx	Edema	Setzer Classification	Czernicki Classification		
						CST	Sensitive P	SCT
1	C5–C6	-	-	C4, C7	3	III	III	III
2	T3	Homogenous	C3–T2	C2, T4	1	NA	NA	NA
3	C4–C5	Tumor walls, cystic portion	C7–T2	C3, C6	2	II	II	II
4	C7	Homogenous	C6	-	2	I	II	II
5	C3–C5	Tumor walls, cystic portion	-	C2–C3, and C6–C7	2	I	III	III
6	C5	-	-	-	1	I	I	I
7	C3–C4	-	-	-	1	I	I	I
8	T2	-	-	T2	1	I	I	I

CST: corticospinal tract, NA: not applicable, P: pathways, SCT: spinocerebellar tracts.

## Data Availability

Data are unavailable due to ethical restrictions.

## Supplementary Materials

The following supporting information can be downloaded at https://www.mdpi.com/article/10.3390/cancers16162834/s1, Table S1: Tensor metrics analysis according to the spine level.
