# Supplementary material for: Value of Spinal Cord Diffusion Imaging and Tractography in Providing Predictive Factors for Tumor Resection in Patients with Intramedullary Tumors: A Pilot Study
Source: Cancers (Basel). 2024 Aug 13;16(16):2834. doi: 10.3390/cancers16162834 (PMC11352615; doi:10.3390/cancers16162834)
Supplement: Supplementary file 1 [file cancers-16-02834-s001.zip › cancers-3126777-supplementary.pdf]

**Supplementary Material**

| Subjects | | C1 | C2 | C3 | C4 | C5 | C6 | C7 |
|---|---|---|---|---|---|---|---|---|
| Healthy subjects | FA | 0.57 ± 0.04 | 0.61 ± 0.03 | 0.61 ± 0.04 | 0.58 ± 0.04 | 0.56 ± 0.05 | 0.51 ± 0.08 | 0.46 ± 0.10 |
| (n = 49) | MD | 1.17 ± 0.08 | 1.25 ± 0.09 | 1.24 ± 0.11 | 1.23 ± 0.11 | 1.22 ± 0.13 | 1.25 ± 0.16 | 1.32 ± 0.25 |
| | AD | 1.99 ± 0.14 | 2.21 ± 0.12 | 2.19 ± 0.12 | 2.11 ± 0.13 | 2.06 ± 0.14 | 2.00 ± 0.14 | 2.01 ± 0.25 |
| | RD | 0.75 ± 0.08 | 0.76 ± 0.09 | 0.77 ± 0.12 | 0.78 ± 0.12 | 0.80 ± 0.13 | 0.87 ± 0.18 | 0.97 ± 0.27 |
| Subject 1 | FA | 0.29 | 0.26 | 0.28 | 0.31 | 0.31 | 0.31 | 0.33 |
| | MD | 2.09 | 2.02 | 2.01 | 1.70 | 1.86 | 1.71 | 1.58 |
| | AD | 2.60 | 2.51 | 2.62 | 2.28 | 2.50 | 2.31 | 2.13 |
| | RD | 1.84 | 1.76 | 1.70 | 1.41 | 1.54 | 1.41 | 1.31 |
| Subject 2 | FA | 0.59 | 0.44 | 0.31 | 0.25 | 0.30 | 0.29 | 0.26 |
| | MD | 0.98 | 1.22 | 2.09 | 2.11 | 1.80 | 1.40 | 1.59 |
| | AD | 1.72 | 1.86 | 2.67 | 2.59 | 2.26 | 1.79 | 1.97 |
| | RD | 0.61 | 0.91 | 1.80 | 1.88 | 1.56 | 1.21 | 1.41 |
| Subject 3 | FA | 0.47 | 0.42 | 0.23 | 0.13 | 0.13 | 0.22 | 0.20 |
| | MD | 1.28 | 1.48 | 1.61 | 2.20 | 2.22 | 1.43 | 1.78 |
| | AD | 1.95 | 2.17 | 2.01 | 2.46 | 2.48 | 1.73 | 2.10 |
| | RD | 0.95 | 1.14 | 1.41 | 2.07 | 2.09 | 1.29 | 1.62 |
| Subject 4 | FA | 0.56 | 0.54 | 0.60 | 0.54 | 0.48 | 0.37 | 0.24 |
| | MD | 1.06 | 1.39 | 1.19 | 1.30 | 1.47 | 1.62 | 1.60 |
| | AD | 1.83 | 2.30 | 2.11 | 2.18 | 2.31 | 2.30 | 2.01 |
| | RD | 0.71 | 0.94 | 0.73 | 0.86 | 1.05 | 1.28 | 1.40 |
| Subject 5 | FA | 0.35 | 0.20 | 0.17 | 0.12 | 0.11 | 0.16 | 0.17 |
| | MD | 1.42 | 1.77 | 1.92 | 2.50 | 2.44 | 1.85 | 1.71 |
| | AD | 1.97 | 2.16 | 2.28 | 2.79 | 2.70 | 2.14 | 1.96 |
| | RD | 1.15 | 1.57 | 1.74 | 2.36 | 2.31 | 1.71 | 1.58 |
| Subject 6 | FA | 0.57 | 0.55 | 0.59 | 0.50 | 0.33 | 0.48 | 0.33 |
| | MD | 1.04 | 1.45 | 1.28 | 1.39 | 1.55 | 0.84 | 1.41 |
| | AD | 1.78 | 2.41 | 2.19 | 2.17 | 2.08 | 1.31 | 1.94 |
| | RD | 0.68 | 0.98 | 0.82 | 0.99 | 1.29 | 0.62 | 1.15 |
| Subject 7 | FA | 0.53 | 0.62 | 0.54 | 0.53 | 0.63 | 0.57 | 0.58 |
| | MD | 1.23 | 1.40 | 1.30 | 1.51 | 1.12 | 1.08 | 1.21 |
| | AD | 2.20 | 2.31 | 2.31 | 2.41 | 2.06 | 1.81 | 2.14 |
| | RD | 0.81 | 0.85 | 0.79 | 1.06 | 0.65 | 0.71 | 0.75 |
| Subject 8 | FA | 0.62 | 0.63 | 0.64 | 0.62 | 0.61 | 0.61 | 0.53 |
| | MD | 0.92 | 1.09 | 1.12 | 1.18 | 1.05 | 1.06 | 1.06 |

| | | | | | | | |
|---|---|---|---|---|---|---|---|
| AD | 1.69 | 1.99 | 2.05 | 2.08 | 1.90 | 1.94 | 1.77 |
| RD | 0.53 | 0.64 | 0.65 | 0.73 | 0.63 | 0.63 | 0.70 |

AD: axial diffusivity, FA: fractional anisotropy, MD: mean diffusivity, RD: radial diffusivity
Note that for healthy subjects, results were represented as mean ± standard deviation.
